# The ears of the African elephant: unexpected high seroprevalence of *Plasmodium ovale* and *Plasmodium malariae* in healthy populations in Western Africa

**DOI:** 10.1186/1475-2875-13-240

**Published:** 2014-06-18

**Authors:** Cécile Doderer-Lang, Pascal S Atchade, Lydia Meckert, Elodie Haar, Sylvie Perrotey, Denis Filisetti, Ahmed Aboubacar, Alexander W Pfaff, Julie Brunet, Nicodème W Chabi, Casimir D Akpovi, Ludovic Anani, André Bigot, Ambaliou Sanni, Ermanno Candolfi

**Affiliations:** 1 Institut de Parasitologie et de Pathologie Tropicale Université de Strasbourg, 3, Rue Koeberlé, F67000 Strasbourg, France; 2 Laboratoire de Biochimie et de Biologie Moléculaire, Université d’Abomey Calavi, 04 BP 0320 Cotonou, Bénin; 3 Agence Nationale pour la Transfusion Sanguine (Ministère de la Santé), 01 B.P. 511 Cotonou, Bénin

**Keywords:** *Plasmodium*, Seroprevalence, Recombinant protein, ELISA, Africa

## Abstract

**Background:**

Malaria Is A Life-Threatening Pathology In Africa. *Plasmodium Falciparum* And *Plasmodium Vivax* Attract The Most Focus Because Of Their High Prevalence And Mortality. Knowledge About The Prevalence Of The Cryptic Pathogens *Plasmodium Ovale* And *Plasmodium Malariae* Is Limited. Thanks To Recombinant Tools, Their Seroprevalence Was Measured For The First Time, As Well As The Prevalence Of Mixed Infections In A Malaria-Asymptomatic Population In Benin, A Malaria-Endemic Country.

**Methods:**

A Panel Of 1,235 Blood Donations Collected Over Ten Months In Benin Was Used For Validation Of The Recombinant Tools. Recombinant *P. Falciparum*, *P. Malariae*, *P. Ovale* MSP1, And *P. Falciparum* AMA1 Were Engineered And Validated On A Biobank With Malaria-Infected Patients (N = 144) Using A Species-Speific ELISA Test (Recelisa). Results Were Compared To An ELISA Using A Native *P. Falciparum* Antigen (NatELISA).

**Results:**

Among Microscopically Negative African Blood Donors, 85% (1,050/1,235) Present Antibodies Directed To Native *P. Falciparum,* 94.4% (1,166/1,235) To r*Pf*MSP1 And r*Pf*AMA1, 56.8% (702/1,235) To r*Po*MSP1, 67.5% (834/1235) To r*Pm*MSP1 And 45.3% Of The Malaria Seropositive Population Had Antibodies Recognizing The Three Species.

**Conclusion:**

A High Rate Of Antibodies Against *P. Ovale* And *P. Malariae* Was Found In Asymptomatic Blood Donors. The Proportion Of Mixed Infections Involving Three Species Was Also Unexpected. These Data Suggest That Determining Seroprevalence For These Cryptic Species Is An Appropriate Tool To Estimate Their Incidence, At The Eve Of Upcoming Anti-*P. Falciparum* Vaccination Campaigns.

## Background

Malaria is one of the most life-threatening diseases in the world. In 2010, 219 million cases were reported globally [1], leading to 660,000 deaths, mostly of children under five years old. Five parasitic species cause human malaria (*Plasmodium falciparum*, *Plasmodium vivax, Plasmodium malariae*, *Plasmodium ovale*, and *Plasmodium knowlesi*), which are transmitted by the bite of infected *Anopheles* mosquitoes. Despite huge efforts to control the disease, resurgence has been observed in many countries due to climate instability, global warming, civil disturbances, drug resistance, and increasing travel between endemic and non-endemic areas [2].

Identifying the most affected countries’ direct resources and validating control measures is essential to reducing malaria’s incidence (target: 75% by 2015) [1]. Epidemiological surveillance seeks to assess malaria’s prevalence over time and identify the species’ geographical distribution. Vaccines against *P. falciparum* and *P. vivax* are in progress [3]; not so for *P. malariae* and *P. ovale*, where prevalence has been insufficiently investigated. To further complicate matters, low levels of parasitaemia and mixed infections can affect diagnosis of *P. malariae* and *P. ovale*. Birkenmeyer *et al.* recently sequenced genes encoding major erythrocyte stage markers of *P. malariae* and *P. ovale*, demonstrating the feasibility of Merozoite Surface Protein 1 (MSP1) recombinant production [4] and use in ELISA to detect antibodies against *P. malariae* and *P. ovale*[5]. Proteins were selected according to their ability to elicit antibody production and chosen from among previously described vaccine candidates. MSP1 is one of the main surface proteins at the merozoite stage expressed in all *Plasmodium* species and plays a role in red blood cell (RBC) invasions [6]. *Plasmodium falciparum* AMA1 is a blood-stage antigen that aids in orienting the merozoite during invasion of RBC. Anti-AMA1 antibodies tend to be present in individuals who have acquired natural immunity [7].

Estimation of malaria prevalence is historically done by optical microscopy but a sensitivity of 50 parasites/μL is insufficient [8]. Further, highly trained staff is necessary, rendering this approach unsuitable for large-scale monitoring. Rapid diagnostic tests and PCR methods are also inappropriate for broad evaluation. ELISA antigen detection of *Plasmodium* lactate dehydrogenase (pLDH) has been documented as a valuable tool for assessing prevalence in a blood donor population [9]. However, detectability is limited to one parasite/μL and the assay is inappropriate for *P. malariae* and *P. ovale* identification*.* Furthermore, various factors influence the direct detection of parasites, among them parasite clearance due to acquired immunity, drug treatment, season variability and sporadic transmission in low-transmission areas. For this reason, seroprevalence measurement has been explored as an accurate tool for estimating transmission intensity and the potential effects of any measures used to control (and ultimately eliminate) malaria [10]. Indeed, antibodies against the four *Plasmodium* species appear within days or weeks of erythrocyte invasion, and can persist for months or years reflecting exposure to the parasites [11].

Immunofluorescence detection of malaria antibodies was until recently the gold standard [12], but is unsuitable for high-throughput screening. ELISA-based seroprevalence screening is a potentially useful epidemiological tool [13]. An immuno-enzymatic assay combining the crude *P. falciparum* antigen and recombinant *P. vivax* proteins was already developed, exhibiting high specificity and analytical sensitivity (96.7 and 93.1%, respectively) in the detection of *Plasmodium* antibodies [14]. However, this technique could not discriminate between the four species.

In this work, the identification and production of recombinant proteins from *P. falciparum*, *P. ovale* and *P. malariae* was reported to establish an ELISA test for the detection of *Plasmodium* species-specific antibodies. Immunoassay performances were first assessed in a population of *Plasmodium*-infected French travellers. For the first time, the distribution of the *P. malariae* and *P. ovale* in endemic malaria areas in Benin (Western Africa) was evaluated in a blood donor population.

## Methods

### Samples from *Plasmodium*-infected patients for sensitivity calculation

Malaria patients returning from endemic countries sera (n = 144), diagnosed and referred at the Strasbourg University Hospital were used for the study. All patients reported fever and were microscopically diagnosed positive for *P. falciparum* (n = 106), *P. malariae* (n = 12), or *P. ovale* (n = 26). All results were confirmed by PCR. Every patient was treated and the samples anonymized. This population was used to determine the recombinant ELISA assay’s clinical sensitivity and positive predictive value.

### Negative samples

Blood donor samples were collected at the Etablissement Français du Sang d’Alsace (EFS Alsace). Donors were classified as unexposed-to-malaria (192 samples) if their questionnaire responses indicated never having travelled to an endemic area. These samples were used to calculate the test’s specificity and negative predictive value.

### Samples from Beninese blood donors

Plasma and total blood samples from blood donors without apparent malaria symptoms (n = 1,235) were collected over ten months (May 2009 to February 2010) in six Beninese departmental blood centres [9]. Each donor signed a consent form, and both the Direction of Benin National Blood Transfusion Agency and the Research Ethics Committee of the Republic of Benin validated the protocol. The collection period was divided into a long rainy season (LRS) from May to July (n = 387), a short dry season (SDS) from August to September (n = 217), a short rainy season (SRS) from October to November (n = 408), and a long dry season (LDS) from December to February (n = 223). Two expert biologists performed parasitic examinations on all samples via microscopy on a May-Grünwald-Giemsa-stained thin and thick film. Positivity for Plasmodium was detected microscopically in 290 donors, who were used to assess the performance of the recombinant proteins. ELISA tests using a native *P. falciparum* antigen and recombinant proteins from *P. falciparum*, *P. ovale* and *P. malariae* were used to assess seroprevalence in the Beninese samples. Five donors gave their blood twice in two different seasons (LRS and SRS); they were kept due to the low number of repeat donors, since they were positive for malaria antibodies.

### Recombinant proteins

Nucleotide constructs encoding 373 AA (amino acids) of *P. falciparum* MSP1 (Accession Number XP_001352170.1), 356 AA of *P. ovale* MSP1 (ACZ51239.1), and 350 AA of *P. malariae* MSP1 (ACZ51237.1) corresponding to the C-terminus region of the protein (42 kD) and a nucleotide construct encoding 448 AA of *P. falciparum* AMA1 (XP_001348015.1) were commercially synthesized with an *Escherichia coli* codon bias (Genscript, Piscataway, NJ, USA). Consensus sequences derived from the alignment of several strains for each species and each protein were used for the design of synthetic genes. All genes were inserted in an expression vector containing a Maltose binding protein fusion partner pMAL-c2X® (New England BioLabs, Ipswich, MA, USA), produced in *E. coli* expression hosts and purified on amylose resin and DEAEsepharose ®(GE healthcare, Uppsala, Sweden).

### *Plasmodium* species DNA detection by PCR

Detection of *P. falciparum* based on the amplification of the STEVOR gene, a subtelomeric multiple copy gene, was performed according to Filisetti *et al.*[15]. The other infecting *Plasmodium* species were identified using a genus and species-specific nested PCR, in addition to a PCR tailored to a specific *P. ovale* subspecies (*P. ovale wallikeri*) [16].

### ELISA using *Plasmodium falciparum* native antigen (NatELISA) and recombinant protein (RecELISA)

Antibody screening was performed using an in-house ELISA test derived from a commercial assay (DiaMed) [14]. Native *P. falciparum* antigen produced from *in vitro* culture (NatELISA) [17] or recombinant rMSP1 for *P. ovale*, *P. malariae*, *P. falciparum* and r*P. falciparum* AMA1 (RecELISA) were immobilized on 96-well plates overnight at 4°C in coating buffer and blocked for one hour with PBST (Phosphate Buffered Saline with Tween 0.05%) containing 1% BSA (bovine serum albumin) (Merck, Darmstadt, Germany). After washing with PBST, 200 μl of diluent buffer (PBST with BSA 0.1%) were dispensed into each well and 10 μl of serum were incubated for one hour at 37°C. On the same plate, 10 μL of positive controls specific for each species and negative controls in triplicate were added. After three washes with PBST, 100 μl of horseradish peroxidase labelled monoclonal rabbit anti human IgG (Sigma-Aldrich, St Quentin, France) were incubated for 30 min at 37°C. After three washes with PBST, 100 μl of TMB plus substrate solution (Tetramethylbenzidine) (Kem-en-tec, Denmark) were incubated for 15 min at 37°C and the reaction was stopped with 50 μl of 0.5 M sulphuric acid. The absorbance was read within 30 min at 450 nm against 620 nm. Test validation required the positive OD to be >0.500 and the negative OD <0.200. The cut-off value was set at three-fold the negative control wells’ average OD. The antibody (Ab) index of each sample was defined as ratio of its OD value and the cut-off value. The sample was considered positive if the Ab index was >0.7, and negative if the Ab index was ≤0.7.

All recombinant assays were performed on an EVOLIS Microplate System (Bio-Rad) at the Department of Microbiology of the Hôpitaux Universitaires de Strasbourg.

### Statistical analysis

Statistical differences between *P. falciparum* native antigen ELISA (NatELISA) and recombinant ELISA (RecELISA) performances were analysed using Chi-squared and t-tests, as detailed in the Results section, below. Significant differences in antibody prevalence depending on the season, observed during follow-up, were assessed using the Chi-squared test. A p-value of less than 0.05 was considered significant. Correlation between antibody titre and ELISA index for the recombinant antigens was calculated using the Pearson correlation test. (GraphPad Prism 6.0 software, La Jolla, CA, USA).

## Results

### Performance of the RecELISAs

The assay was first evaluated on 144 plasma samples from malaria patients diagnosed using microscopy and confirmed by PCR. The RecELISA performed better than the NatELISA, which used a *P. falciparum* native antigen (Table 1). Recombinant proteins from other *Plasmodium* species presented a good sensitivity; only three of 26 *P. ovale* cases were missed, and every *P. malariae* infection was detected. The combined performance of the three RecELISAs (using recombinant proteins from three species) was superior to that of the NatELISA (Table 2). On the other hand, the RecELISA failed to detect nine positive cases, most of them also missed by the NatELISA (Table 3). Three of the nine missed infections were *P. ovale* cases, while six were *P. falciparum*. However, eight cases were detected exclusively by RecELISA. Four of those were *P. ovale* and one was *P. malariae*; the three others were *P. falciparum* infections. Overall specificity was 97.9%, calculated on 192 unexposed to malaria blood donors.

**Table 1:** **Evaluation of the performance of ELISA using species-specific recombinant *Plasmodium *proteins (RecELISA) in comparison to an ELISA using *P. falciparum *native antigen (NatELISA) in a population of malaria-infected travellers (n = 144): 106 *Plasmodium falciparum *patients; 26 *Plasmodium ovale *patients and 12 *Plasmodium malariae *patients**

**Species**	**Microscopy + PCR**^**a **^**positive**	**NatELISA pos**	**RecELISA r *Pf *MSP1 + r *Pf *AMA1 pos**	**RecELISA r *Po *MSP1 pos**	**RecELISA r *Pm *MSP1 pos**	**RecELISA Total pos**
***P. falciparum***	106/144	98/106 (92.5%)	100/106	ND	ND	135/144^c^
***P. ovale***	26/144	20/26 (76.9%)	ND	23/26	ND	
***P. malariae***	12/144	11/12 (91.7%)	ND	ND	12/12	
**Negative**	ND^b^	5/192	3/192	3/192	3/192	4/192

For the calculation of specificity, blood donors not exposed to malaria (n = 192) were used exposed to malaria (n = 192) were used.

^a^each sample was confirmed by PCR.

^b^ND: not done. Blood donors not travelling in malaria-endemic countries were considered as negative (n = 192).

^c^results for the combined recombinant for the three species.

**Table 2:** Recapitulative performances of the assay

**Performances**	**NatELISA**	**RecELISA r *Pf *MSP1 + r *Pf *AMA1**	**RecELISA r *Po *MSP1**	**RecELISA r *Pm *MSP1**	**RecELISA Total**
**Sensitivity**	129/144 (89.6%)	100/106 (94.3%)	23/26 (88.5%)	12/12 (100%)	135/144 (93.8%)
**Specificity**	187/192 (97.4%)	189/192 (98.4%)	189/192 (98.4%)	189/192 (98.4%)	188/192 (97.9%)
**PPV**	96.3%	97.1%	88.5%	80%	97.1%
**NPV**	92.6%	96.9%	98.4%	100%	95.4%

**Table 3:** **Performance of *Plasmodium falciparum *native antigen ELISA (NatELISA) *versus *three specific recombinant *Plasmodium *proteins ELISA (RecELISA Total)**

	**RecELISA Total Pos**	**RecELISA Total neg**	**Total**
**NatELISA pos**	127	2	129
**NatELISA neg**	8	7	15
**Total**	**135**	**9**	**144**

Total comparative results for malaria-infected patients (n =144) microscopically diagnosed in a non-endemic country.

Positive RecELISA Total *versus* positive RecELISA: 135/144 *versus* 129/144: Chi-square test, p = 0.2008.

### Antibody prevalence in Beninese blood donors

**Figure 1:**
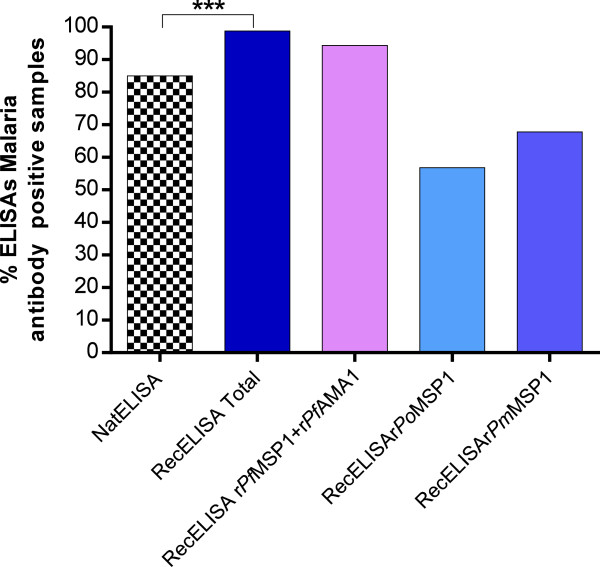
**Seroprevalence of antibodies detected by native *P. falciparum *** **or a set of recombinant proteins from Plasmodium (r *Pf *** **AMA1 and rMSP1 from *P. falciparum*, *P. ovale *** **and *P. malariae*) in 1,235 Beninese blood donors.**

**Figure 2:**
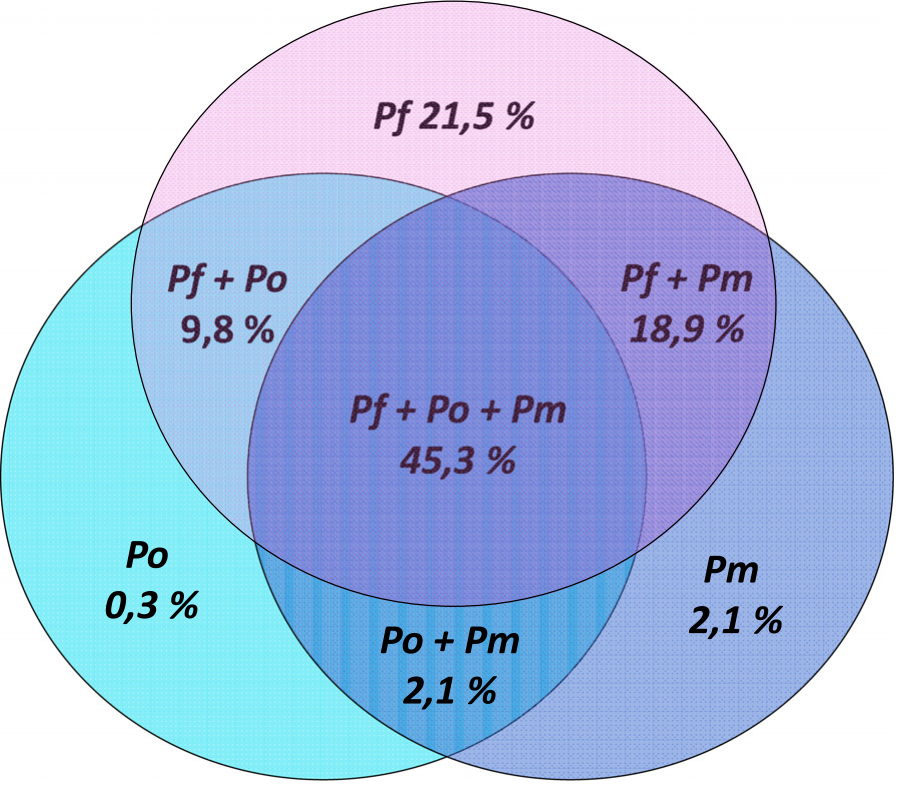
Venn diagram showing a distribution of mixed malaria infections among 1,220 malaria antibody-positive Beninese blood donors.

The recombinant antigens were used to evaluate species-specific antibody prevalence in a panel of Beninese blood donors. A population of blood donors with microscopically detectable *Plasmodium* infections was isolated from a previous study [9]. Anti-*P. falciparum* antibodies were detected in almost 90% (259/290) of the population with the NatELISA. Only two mixed infections (*P. malariae* and *P. falciparum*) were detected by microscopy, confirmed by PCR, and positive with all recombinant proteins (Table 4). Combining the data from the three assays (RecELISA Total) reveals that almost all of the investigated population had antibodies against *P. falciparum*, *P. ovale* and *P. malariae*, suggesting a non-negligible presence of co-infection (Table 4). In all blood donors, 85% (1,050/1,235) presented antibodies directed to native *P. falciparum* antigen, 94.4% (1,166/1,235) to *P. falciparum* recombinant r*Pf*MSP1 and r*Pf*AMA1, 56.8% (702/1,235) to the *P. ovale* recombinant r*Po*MSP1, and 67.5% (834/1,235) to the *P. malariae* recombinant r*Pm*MSP1. By summing up all the patients bearing antibodies directed to recombinant proteins, the prevalence was 98.8% (1,220/1,235) (Figure 1). These results demonstrate that the exclusive use of a *P. falciparum* native antigen leads to an underestimation of the seroprevalence of *Plasmodium* infection (Table 5). The 173 donors positive under RecELISA and negative under NatELISA were divided into seven populations depending on the nature of the recombinant protein detected. Notably, almost 30% of that population was positive for *P. falciparum*, *P. ovale* and *P. malariae* (Table 6). In the RecELISA malaria antibodies-positive population (n = 1,220), 45.3% of the sera had antibodies recognizing recombinant proteins from all three species (Figure 2). According to this assay, 76.1% of the population were suspected to have an infection with at least two different species of *Plasmodium*.

**Table 4:** **Evaluation of the seroprevalence of three *Plasmodium *species in blood donors from Benin by the three specific recombinant *Plasmodium *proteins ELISA (RecELISA) and *Plasmodium falciparum *native antigen ELISA (NatELISA) in a population of asymptomatic Benin blood donors with positive parasitaemia (n = 290): 288 *Plasmodium falciparum *and two mixed infection *Plasmodium falciparum and Plasmodium malariae *identified by microscopy**

**Results of microscopical examination**	**NatELISA**	**RecELISA r *Pf *MSP1 + r *Pf *AMA1**	**RecELISA r *Po *MSP1**	**RecELISA r *Pm *MSP1**	**RecELISA Total**
288 *P. falciparum*	257/288 (89.2%)	282/288 (97.9%)	195/288 (67.7%)	229/288 (79.9%)	287/288 (99.6%)
2 *P. malariae* and *P. falciparum*	2/2 (100%)	2/2 (100%)	2/2 (100%)	2/2 (100%)	2/2 (100%)

**Table 5:** **Comparative results of *Plasmodium falciparum *native antigen ELISA (NatELISA) and three specific recombinant *Plasmodium *proteins ELISA (RecELISA Total) for Beninese blood donors (n = 1,235)**

	**RecELISA Total Pos**	**RecELISA Total Neg**	**Total**
**NatELISA pos**	1047	3	1050
**NatELISA neg**	173	12	185
**Total**	**1220**	**15**	**1235**

Positive RecELISA Total *versus* positive NatELISA: 1,220/1,235 *versus* 1,050/1,235: Chi-square test, p < 0.0001.

**Table 6:** **Distribution of species in the population negative for the *Plasmodium falciparum *native antigen ELISA (NatELISA) and positive for the three specific recombinant *Plasmodium *proteins ELISA (RecELISA Total)**

**Species**	**RecELISATotal pos**	**Percentage**
***Pf***	65	37.6%
***Pm***	6	3.5%
***Po***	3	1.7%
***Po*** **+** ***Pf***	20	11.6%
***Po*** **+** ***Pm***	3	1.7%
***Pm*** **+** ***Pf***	28	16.2%
***Pf*** **+** ***Po*** **+** ***Pm***	48	**27.7%**
**Total**	173	

**Figure 3:**
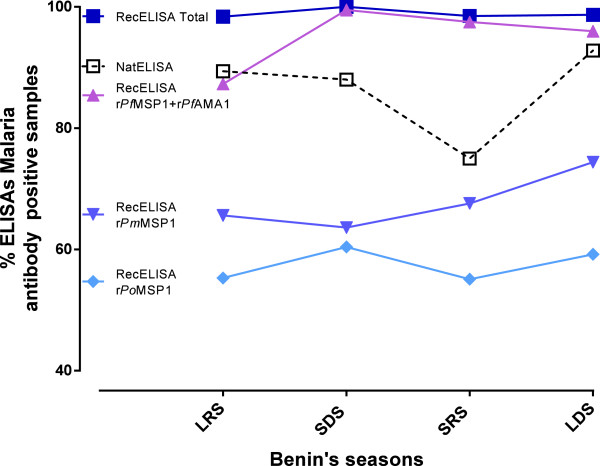
**Seasonal evolution of seroprevalence for antibodies detected by native *P. falciparum *** **antigen and recombinant proteins r** ***Pf*** **AMA1 + r** ***Pf*** **MSP1, r** ***Po*** **MSP1, and r** ***Pm*** **MSP1 in 1,235 malaria blood donors in Benin; LRS: long rainy season (May to July); SDS: short dry season (August to September); SRS: short rainy season (October to November); LDS: long dry season (December to March).**

**Figure 4:**
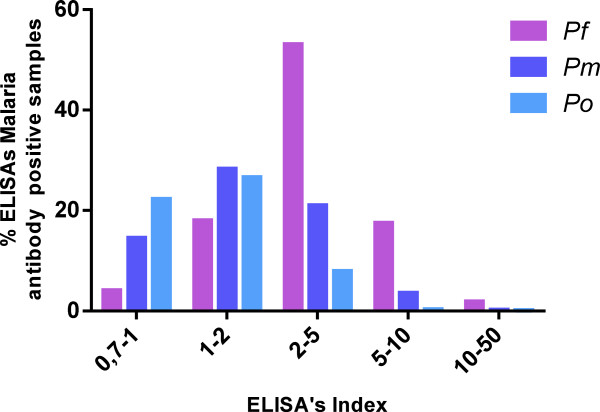
**Distribution of positive samples according to the ELISA’s index for *P. falciparum*, *P. malaria *and *P. ovale *recombinant antigens.** The cut-off value was calculated by multiplying the negative control wells’ average optical density (OD) by three. The antibody (Ab) index of each sample was calculated by dividing its OD value by the cut-off value. The sample was considered positive if the Ab index was >0.7, and negative if the Ab index was ≤0.7.

Seroprevalence of antibodies directed to the three species throughout the four seasons of Benin’s semi-equatorial climate (Figure 3) was investigated. Recognition of antibodies directed to *P. malariae* and *P. ovale* rose until the end of the LDS. Finally, summing up the data from the three species, the rate of antibodies was constant throughout the year (Figure 3). Combining the three species’ recombinant proteins improved sensitivity by 10% over the use of recombinant proteins from *P. falciparum* alone. The prevalence of antibodies against the native *P. falciparum* antigen dropped significantly at the SRS, and then increased at the LDS. Distribution of positive samples according to ELISA index for *P. falciparum*, *P. malariae* and *P. ovale* recombinant antigens was also estimated and showed low rates for antibodies directed to *P. ovale* and *P. malariae* species and higher rates for antibodies directed to *P. falciparum* (Figure 4). No correlation between parasite density and antibody titre for NatELISA and RecELISA for the three species was observed.

## Discussion

The antigenicity of these recombinant proteins was first evaluated in patients infected by *P. falciparum, P. ovale* and *P. malariae* in European settings. The analytical parameters (sensitivity, specificity, PPV, and NPV) using recombinant proteins were better than those of an ELISA using a native antigen from *P. falciparum*. In particular, the use of recombinant proteins from the *P. ovale* and *P. malariae* species largely enhanced detection rates for those infections. The efficacy of these new tools was optimized using synthetic genes integrating consensus sequences available in GenBank [4,5], covering gene polymorphisms between known strains of each species and optimized for expression in a prokaryotic system. The biobank used for validation of the reagent involved sera from patients specifically infected by one of the three species of pathogens. Specificity was high in all cases, reaching an overall level of 97.9%. In general, the use of various recombinant antigens resulted in better sensitivity without sacrificing specificity. However, this technology should be further improved, especially for *P. ovale*. The surface antigen MSP1 is highly polymorphic due to wide strain variability. This pitfall in the use of recombinant proteins leading to the presentation of limited epitopes to humoral response was previously highlighted [18]. A combined analysis of more sequences from *P. ovale* strains, now accessible on GenBank, might resolve this problem.

The RecELISA’s strong performance allowed the study of Beninese blood donors. Recent studies using microscopic examination in southern Benin have demonstrated the presence of mixed infection at the following rates: 2% *P. ovale* and 3.1% *P. malariae*, associated or not to *P. falciparum:* 1.2% *P. falciparum*/*P. malariae*, 2.4% *P. falciparum*/*P. ovale* and 0.1% *P. falciparum*/*P. malariae*/*P. ovale*[19]. In this study, in a population of 1,235 healthy blood donors, microscopic examination revealed 21.2% *P. falciparum* and 0.15% *P. falciparum*/*P. malariae*. Similarly, in Burkina Faso, microscopy detected 72.5% *P. falciparum*, 13.2% *P. malariae* and 1.8% *P. ovale* in a population of 830 children between three and 15 years old, of which 18.8% were febrile [20]. Microscopy probably underestimates prevalence, especially for the cryptic species with low parasitaemia, *P. ovale* and *P. malariae*. This diagnostic tool has a detection threshold of five to 50 parasites/μL [8], while PCR reaches 0.001 p/μL [15]. A recent review [21] showed that the use of molecular methods such as PCR, facilitates greatly increased *P. malariae* and *P. ovale* detection sensitivity. Snounou *et al.*, using PCR, were able to detect in a microscopically negative Bissau-Guinean population, 23.3% *P. malariae* and 6.9% *P. ovale*[22] infection. However, submicroscopic and even sub-PCR infections do exist and can only be revealed with serology [11]. Therefore, delimiting transmission areas is essential [10]. Thus, seroprevalence reflects exposure, overcoming sampling bias associated with seasonal and year-to-year variation [11].

However, seroprevalence was exclusively tested using *P. falciparum* antigens, neglecting *P. ovale* and *P. malariae*. The use of recombinant proteins of *P. malariae* and *P. ovale* allowed revealing high prevalence of antibodies directed to these cryptic parasites. In Benin, an increased production of antibodies directed to *P. ovale* and *P. malariae* was observed until the LDS. This may be due to the delayed appearance of these species after the first infection, as has already been observed [23]. Using recombinant proteins from *P. falciparum* (MSP1 and AMA1), seroprevalence increased after the LRS and maintained high levels until the LDS, reflecting the long-lasting presence of IgG directed to r*Pf*AMA1 and r*Pf*MSP1 antigens. Similarly, the seroprevalence against *P. falciparum* recombinant proteins did not vary between wet and dry seasons in the Gambia [24]. Interestingly, a drop in the recognition of a native *P. falciparum* antigen was observed during the SRS. This may be due to a deficit of MSP1 and AMA1 in the native antigen, prepared from a synchronized culture in which an 80% schizont proportion shifted the recognition of the malarial antibodies towards the detection of older stages of *P. falciparum*. Another hypothesis is that the variation in antibody response is due to differences in antigen concentration and relative proportions of MSP1 in native and recombinant antigenic preparations.

This work shows that more than half of the studied population was in contact with other species of *Plasmodium* in addition to *P. falciparum*, and that 76% of the investigated healthy population were exposed to more than one species. These results indicate that the prevalence of non-falciparum species is much higher than previously estimated. The levels of antibodies directed to these two cryptic species is low, possibly due to their parasitaemia levels. On the contrary, high levels of antibodies against *P. falciparum* are observed, corresponding to high parasitaemia levels.

While *P. falciparum* infection remains the primary concern, the detection of *P. ovale* and *P. malariae* is still important. Although both cause mild infection, *P. malariae* can cause chronic nephritic syndrome, leading to adverse reactions during treatment and a high rate of mortality [25]. There is also a risk of recurrence decades after initial exposure, even when the infected population has left the endemic region [26]. Immune protection against *P. falciparum* is not fully effective against *P. malariae*[27]. Furthermore, *P. malariae* is more prevalent when transmission and infection rates of *P. falciparum* are lower, probably because of negative interactions with *P. falciparum*; similar interactions with *P. ovale* might exist [28]. Positive selection of anti-*P. falciparum* medication caused the prevalence of *P. malariae* to rise for four years in Burkina Faso. The authors predicted that in the absence of *P. falciparum* (ie, due to the effects of a vaccine directed against *P. falciparum*), *P. malariae* parasitaemia and gametocyte carriage could increase and maintain severe malaria infection [20].

## Conclusion

Given the advent of vaccine campaigns against *P. falciparum*, other species must be monitored more closely as their presence will remain a serious health concern for exposed populations in endemic countries [29].

In conclusion, with the elimination of malaria back on the agendas of world agencies, it is necessary to monitor the effects of anti-malarial measures. In this work a standardized, sensitive and specific tool was developed that facilitates measuring and monitoring the transmission intensity of three *Plasmodium* species. Antibody prevalence for all species of *Plasmodium* will permit detection of residual infections and variations in malaria transmission. Of course, more complex combinations of recombinant *Plasmodium* proteins should be used in future studies to avoid the disadvantages of antigenic polymorphism and variable individual responsiveness, especially for *P. ovale*. These kinds of recombinant tools could also be used to enhance blood transmission safety in non-endemic regions [30].

## Competing interests

The authors declare that they have no competing interests.

## Authors’ contributions

CDL, PA, AS, and EC designed the study, analysed the data and prepared the report; AS and EC coordinated the study; CDL and PA led the study in each site; LM produced the recombinant proteins; EH, SP, DF, AA, AWP, and JB processed the samples and collected the data in France; NWC, CDA and LA collected the data in Africa. All authors contributed to correcting the report. All authors read and approved the final manuscript.
